# Neighborhood disadvantage and the sales of unhealthy products: alcohol, tobacco and unhealthy snack food

**DOI:** 10.1186/s12889-021-11442-z

**Published:** 2021-07-09

**Authors:** Lauren A. Wallace, Rajib Paul, Shafie Gholizadeh, Wlodek Zadrozny, Caitlan Webster, Melanie Mayfield, Elizabeth F. Racine

**Affiliations:** 1 Department of Public Health Sciences, University of North Carolina at Charlotte, 9201 University City Blvd, Charlotte, NC 28223 USA; 2 College of Computing and Informatics, University of North Carolina at Charlotte, 9201 University City Blvd, Charlotte, NC 28223 USA

**Keywords:** Neighborhood disadvantage, Chronic disease, Social environment, Environmental factors

## Abstract

**Background:**

Individuals may use unhealthy coping mechanisms such as alcohol, tobacco, and unhealthy snack consumption. The purpose of this study was to assess how neighborhood disadvantage is associated with sales of alcohol, tobacco, and unhealthy snacks at stores of a discount variety store chain.

**Methods:**

Alcohol, tobacco, and unhealthy snack sales were measured monthly for 20 months, 2017–2018, in 16 discount variety stores in the United States. Mixed effects linear regressions adjusted for population size, with store-specific random effects, to examine the relationship of weekly unit sales with three outcome variables and neighborhood disadvantage, measured using the Area Deprivation Index (ADI).

**Results:**

The discount variety stores were located in neighborhoods where the median ADI percentile was 87 [interquartile range 83,89], compared to the median ADI percentile of 50 for all US communities, indicating that the stores were located in substantially disadvantaged neighborhoods. For every 1% increase in ADI, weekly unit sales of unhealthy snack food increased by 43 [95% confidence interval, CI 28–57], and weekly unit sales of tobacco products increased by 11.5 [95% CI 5–18] per store. No significant relationship between neighborhood disadvantage and the weekly unit sales of alcohol products was identified.

**Conclusions:**

The positive relationship between neighborhood disadvantage and the sale of tobacco and snack foods may help explain the pathway between neighborhood disadvantage and poor health outcomes. It would be useful for future research to examine how neighborhood disadvantage influences resident health-related behaviors.

## Background

Researchers have established that disadvantaged neighborhoods with a high concentration of residents with low socio-economic status (SES) often have relatively low average life expectancy [1, 2], poor mental and physical health [3–8], and low school completion rates [9]. The reasons for this are not entirely clear. Using a life course approach, Seabrook and Avison [3] highlight that individuals with differing SES have differing stressors, and differing resources to address those stressors, and that individuals often have “linked lives” with individuals of similar SES. Therefore, if an individual with, say, low SES, has friends, neighbors, and family members who also have low SES, that individual will likely be exposed to the stresses and challenges that her or his family, neighbors, and family members experience (e.g., job loss, food insecurity, lack of health care services, etc.). In addition to stress, the built environment in disadvantaged neighborhoods is often quite different from that of more advantaged neighborhoods. Specifically, disadvantaged neighborhoods are less likely to have infrastructure in place that promotes health, such as food stores with healthy options [10], quality health care services [11], and safe, walkable spaces [12, 13].

One measure of neighborhood disadvantage, the area deprivation index (ADI), was originally developed by Gopal Singh [14], and revised by Amy Kind and colleagues at the University of Wisconsin-Madison [15]. The revised ADI is a validated instrument that has been used by researchers and government agencies such as the Centers for Medicare and Medicaid Services [16]. Recently, a number of studies have been published using the ADI [17–22].

Three unhealthy consumption behaviors are tobacco use [23], alcohol use [24], and eating unhealthy snack foods [25]. All three behaviors are major risk factors for chronic disease and premature death [26]. Given that people with low SES in the US are more prone to tobacco use [27, 28], alcohol use [27, 28], and unhealthy snack food intake [25] than people with higher SES, and that all three risky health behaviors have been identified as mechanisms to cope with stress [29], we would expect the sales of these items to be high in areas with higher neighborhood disadvantage.

Neighborhood deprivation has been associated with risky health behaviors including excessive alcohol consumption, physical inactivity, and high-fat diets [29]; studies have examined the relationship between neighborhood disadvantage and the availability of unhealthy products. A study by Lee et al. [30] found that as the proportion of African American residents increased and median household income decreased, the density of tobacco outlets per 1000 in census tracts increased. Similarly, Datta et al. found that higher levels of neighborhood poverty were associated with higher prevalence of smoking [31]. Other researchers found that residents in areas with more tobacco outlets were more likely to start smoking than residents in other areas, and less likely to quit [32]. There is also evidence that greater availability [33, 34] and acceptability of alcohol (or “neighborhood norms”) are linked to higher alcohol use [35, 36]. Research has also found that living in a disadvantaged neighborhood is associated with less access to high-quality food sources [25], greater access to unhealthy snack foods [37], and greater exposure to unhealthy snack food advertising [38]. Separate from those neighborhood risks, there is evidence that low household SES is associated with greater access to unhealthy products [25, 30, 32–39].

Despite the studies described above, there is still a gap in research that examines whether the greater access to unhealthy products actually results in greater purchasing of these products. Also, while living in a neighborhood with low SES is associated with a number of poor health outcomes, there is limited research identifying the specific chain of events that leads to poor health outcomes. In this study, we address a gap in this area of research by examining the purchase of unhealthy products and the role of neighborhood disadvantage. Specifically, the purpose of this study is to assess how neighborhood disadvantage is associated with the sales of unhealthy products (alcohol, tobacco, and unhealthy snacks) at a chain of discount variety stores. Discount variety stores (DVSs) sell a wide range of products. The items we included in our analysis are described in the “Outcome Measures” section. The specific stores in our study were selected by the DVS chain that made its data available for our study. The stores were located in different neighborhoods.

There have been few studies of the sales of unhealthy products in deprived neighborhoods using actual sales data. There is little research in this area likely because sales data is proprietary, and it is rarely in the best interest of the business to share these data with researchers. Because we had the opportunity to use proprietary sales data, we were able to address this gap in the research by examining actual sales data. From this study, the association between neighborhood deprivation and actual purchasing behavior in places where there is typically limited choice adds new sales-based evidence that sales of tobacco and unhealthy snacks are greater in deprived neighborhoods. We hypothesized that there would be a positive association of neighborhood disadvantage with the sales of unhealthy products.

## Methods

### Data sources

We used four data sources: Discount Variety Store (DVS) sales data, US Census data, neighborhood walkability scores, and ADI scores. A chain of DVSs that tend to operate in low income communities in the United States provided weekly sales data from 16 stores over an 85-week period, from August, 2016 through March, 2018. The majority of the stores were in the Southeastern United States. The Discount Variety Store corporation granted permission for the research team to access and utilize these data. One stipulation of the data use agreement was that the research team would not disclose the name of the corporation. ADI data were obtained from the Department of Medicine, University of Wisconsin [15]. Other neighborhood socio-demographic variables were obtained from US Census American Community Survey (ACS) website based on their 2015 estimates. Walkability scores for each store address were obtained from the website www.WalkScore.com [40].

### Outcome measures

We examined the weekly unit sales per store of three “unhealthy” products: tobacco, alcohol, and unhealthy snack foods. Tobacco products were identified in the DVS sales data. There were 111 products included in the tobacco sales outcome measure. Tobacco product examples included menthol cigarettes, cigarillos, and non-menthol cigarettes. Alcohol products were also identified in the DVS sales data. There were 70 alcohol products included in the alcohol sales outcome measure. Alcohol products examples included malt beverages, beer, and wine. Unhealthy snack foods were identified using criteria adapted from Farley et al. (2009) [41], who identified snack foods in four categories: foods that were included are sugar sweetened beverages (excluding diet or sugar free varieties), salty snacks (chips, popcorn, pretzels, salted nuts, salted meats/beef jerky), cookies & pastries (prepackaged cookies, crackers, doughnuts, pastries, small fruit-filled pies), and candy (chocolates, hard candy, gum). There were 305 products included in the unhealthy snack food sales outcome measure.

### Neighborhood disadvantage measure

We measured neighborhood disadvantage using the ADI discussed above. The ADI ranks census block groups in terms of socioeconomic status (0–100, with 100 as the highest deprivation). It combines seventeen socioeconomic measures (such as percent of the population 25 and up with at least a high school diploma, median family income, and percent of households without a vehicle) from census data using principal component analysis [19]. We used the 2018 ADI, which averaged 5 years of data from the American Community Survey (ACS), 2014–2018. Since ADI measures area deprivation, it is unlikely the scores changed significantly over the course of the 85 weeks examined in our study.

### Additional neighborhood characteristic measures

In addition to neighborhood disadvantage, we examined 3 additional measures that were not represented in the ADI yet are potentially associated with the sales of tobacco, alcohol, and unhealthy snack foods. These measures are: race/ethnicity composition, percent of the population that are children, and walkability.

Race/ethnicity for the census block groups (obtained from the 2015 ACS), was measured as the percent of the census block group population that was Non-Hispanic African American. Previous research finds that consumption of alcohol [8, 42], tobacco [43], and unhealthy foods [44] can differ by race and ethnicity; yet these differences may be confounded by other factors such as socioeconomic status [45]. Also from the 2015 ACS, we obtained information on the percent of the census block group population between the ages 0 to 17 years. We included this measure because childhood snacking and obesity have been increasing [46], and previous research notes differences in snacking behavior among children by socioeconomic status [47]. Finally, we included the walkability score for each store as walkability increases access to neighborhood retailers; research has linked greater access to tobacco retailers with greater tobacco use, although the corresponding results for alcohol access and use have been mixed [48]. The Walk Score measure ranges from 0 to 100 with 100 meaning most walkable [49]; the average walk scores for 141 US cities with a population of 200,000 or more was 48 at the time of this writing [40].

### Statistical analysis

We analyzed the data with descriptive and inferential statistical techniques. Descriptive analyses summarized neighborhood disadvantage and characteristics variables as well as census block group population size and census block group median income, using medians and interquartile ranges. The inferential statistical techniques included mixed effects linear regression (MELR) with unit sales as the outcome measures. In our MELR, we included as independent variables: the national percentile of each block group’s ADI score, percentage of African-American population, percentage of population between 0 and 17 years, and walkability scores. Additionally, block-group population size and the study week number were included as control variables. Store-specific random effects were included to account for variations among stores that were unexplained by the estimated fixed effects; the random effect also provided standard errors that account for the repeated sales measures for each store. We assessed multicollinearity among the fixed effects terms with Variance Inflation Factors (VIFs). We used the R/RStudio version 3.6.1 for all analyses.

## Results

The unit sales of unhealthy products varied among stores, where the median weekly unit sales for tobacco were 412 [interquartile range (IQR) 205, 610], alcohol 339 [IQR 147, 428], and unhealthy snack foods 1378 [IQR 883, 2526] (Table 1). Results for the socio-demographic variables indicate that, in general, the DVSs were located in neighborhoods where the median ADI percentile was 87 [IQR 83, 89] (compared to median ADI percentile of 50 for all US communities). The racial composition of the communities was primarily Non-Hispanic African American, where the median percentage of African-Americans was 74% [IQR 61, 87]; that result differed greatly from the national average, 13% [50]. The median percent of the population comprised of children (ages 0–17) was 25% [IQR 23, 29] (compared to 23% of the US population in 2017 [51]). The median walk score was 48 [IQR 39, 59], indicating that the majority of communities were car dependent [40].

**Table 1:** Neighborhood Socio-Demographic Characteristics of 16 Discount Variety Stores^a^

Neighborhood Characteristics	Store Median [IQR^b^] *N* = 16 stores
ADI national rank percentile^c^	87 [83,89]
Household income (in dollars)	25,827 [20,387, 35,320]
Population Size	1068 [901,1889]
Percent of population that is Non-Hispanic African-American	74 [61,87]
Percent population ages 0–17	25 [23,29]
Walk Score^d^	48 [39,59]

^a^ Neighborhood is defined as the US Census Block Group for household income, percent of African-American households; US Census Tract for percent of households with no vehicle access and percent of households participating in the Supplemental Nutrition Assistance Program, a federal program that provides nutrition benefits to low income individuals and families (SNAP); and store address determined neighborhood Walk Score (www.walkscore.com)

^b^ Interquartile Range (IQR)

^c^ ADI is the Area Deprivation Index measured as a percentile of Census Block Groups throughout the US, where the 99th percentile is the highest level of neighborhood disadvantage

^d^ Walk Score ranged from 0 to 100, with 100 being the most walkable

Multivariate analyses indicated that as the ADI percentile increased, the sales of tobacco and unhealthy snacks in DVSs increased significantly. Specifically, for every 1% increase in ADI, the weekly unit sales of tobacco per store increased by 11.48 (95% Confidence Interval, 95% CI 5.02; 17.94) adjusted for population size and other covariates (Table 2). There was also a negative relationship between the racial composition of the neighborhood and tobacco sales: when the percent of Non-Hispanic African-Americans in a neighborhood increased by 1%, the weekly unit sales for tobacco decreased by 6.16 (95% CI -9.06; − 3.27). Finally, there was a negative relationship between neighborhood walk score and tobacco sales: when the walk score increased by 1%, the weekly unit sales for tobacco decreased by 6.18 (95% CI -10.36; − 2.01).

**Table 2:** Relationship between Area Deprivation Index and Neighborhood^a^ Characteristics and the Number of Tobacco Units Sold on Average by Store per Week

Neighborhood Socio-Demographic Characteristics	Coefficient	*p*-value	95% Confidence Level	
ADI national percentile^b^	11.48	0.02	4.37	18.52
Percent of population that is Non-Hispanic African-American	−6.16	0.01	−9.35	−2.98
Percent population ages 0–17	22.00	0.05	4.35	39.64
Walk score^c^	−6.18	0.03	−10.78	−1.59
Conditional R-squared = 0.85				

^a^ Neighborhood is defined as the US Census Block Group for household income, percent of Non-Hispanic African-American households; US Census Tract for percent of households with no vehicle access and percent of households participating in the Supplemental Nutrition Assistance Program, a federal program that provides nutrition benefits to low income individuals and families (SNAP); and store address determined neighborhood Walk Score (www.walkscore.com)

^b^ ADI is the Area Deprivation Index, measured as a percentile of Census Block Groups throughout the US, where the 99th percentile is the highest level of neighborhood disadvantage

^c^ Walk Score ranged from 0 to 100, with 100 being the most walkable

Note: analyses were controlled for sales week (1–85) and population size; coefficients are not standardized

We did not find a relationship between alcohol sales and neighborhood characteristics (Table 3). We found that of the 16 DVSs, alcohol was sold at only 8 stores, which limited statistical power (degrees of freedom =2).

**Table 3:** Relationship between Area Deprivation Index and Neighborhood Characteristics and the Number of Alcohol Units Sold on Average by Store per Week^a^

Neighborhood Socio-Demographic Characteristics	Coefficient	*p*-value	95% Confidence Interval	
ADI national percentile^b^	−11.34	0.35	−38.60	15.91
Percent of population that is Non-Hispanic African-American	−5.13	0.17	−12.13	1.88
Percent population ages 0–17	21.00	0.26	−18.87	60.79
Walk score^c^	0.49	0.91	−11.01	11.98
Conditional R-squared = 0.87				

^a^ Neighborhood is defined as the US Census Block Group for household income, percent of African-American households; US Census Track for percent of households with no vehicle access and percent of households participating in the Supplemental Nutrition Assistance Program, a federal program that provides nutrition benefits to low income individuals and families (SNAP); and store address determined neighborhood Walk Score (www.walkscore.com)

^b^ ADI is the Area Deprivation Index measured as a percentile of Census Block Groups throughout the US, where the 99th percentile is the highest level of neighborhood disadvantage

^c^ Walk Score ranged from 0 to 100, with 100 being the most walkable

Note: analyses controlled for sales week (1–85) and population size; coefficients are not standardized

For each 1% increase in ADI, the weekly unit sales of unhealthy snack food increased by 42.57 (95% CI 28.13; 57.01) adjusted for population size and other covariates (Table 4). In addition, each 1% increase in the percentage of children age 0–17 living in the census block was associated with increased weekly unit sales of unhealthy snacks of 62.73 (95% CI 26.88; 98.59).

**Table 4:** Relationship between Area Deprivation Index and Neighborhood Characteristics and the Number of Unhealthy Snack Food Units Sold on Average by Store per Week^a^

Neighborhood Socio-Demographic Characteristics	Coefficient	*P*-value	95% Confidence Interval	
ADI national percentile^b^	42.57	< 0.001	26.67	58.45
Percent of population that is African-American	−5.52	0.19	−12.64	1.60
Percent population ages 0–17	62.73	0.02	23.28	102.18
Walk score^c^	−7.35	0.22	−17.61	2.92
Conditional R-squared = 0.64				

^a^ Neighborhood is defined as the US Census Block Group for household income, percent of African-American households; US Census Tract for percent of households with no vehicle access and percent of households participating in the Supplemental Nutrition Assistance Program, a federal program that provides nutrition benefits to low income individuals and families (SNAP); and store address determined neighborhood Walk Score (www.walkscore.com)

^b^ ADI is the Area Deprivation Index measured as a percentile of Census Block Groups throughout the US, where the 99th percentile being the highest level of neighborhood disadvantage

^c^ Walk Score ranged from 0 to 100 with 100 being the most walkable

Note: analyses controlled for sales week (1–85) and population size; coefficients were not standardized

## Discussion

We used sales data from a small-format national DVS chain and neighborhood characteristic measures to examine the relationship between neighborhood disadvantage and sales of unhealthy products (tobacco, alcohol, and unhealthy snack foods). Tobacco sales were greater in neighborhoods with greater disadvantage. This may be due to more tobacco advertising in lower SES neighborhoods [52, 53], higher levels of stress among residents [54, 55], and/or greater access to tobacco products [30, 32]. Also, as neighborhood deprivation increased, the sales of unhealthy snack foods increased [44, 47]. Similar to tobacco sales this may be due to increased advertising for unhealthy snack foods found in lower SES neighborhoods [38], increased levels of stress among residents [39], and/or increased access to unhealthy snack products in these communities [37]. There is also evidence that many people with lower income in the United States deliberately choose high fat foods to fulfill caloric needs, a factor that may have contributed to the results [56].

We did not find a relationship between neighborhood characteristics and alcohol sales. However, this result may be due in part to limitations of our data: only 8 of the 16 stores sold alcohol, which limited our statistical power. There were generally no clear differences between the neighborhoods in which DVS locations chose to sell alcohol and those in DVS locations did not. The decision to sell alcohol in these DVSs may be dependent on factors such as store or local alcohol sale policies or local opposition to alcohol sales, although the DVSs in this study were located in states and municipalities that allowed alcohol sales in DVSs. Our study focused on stores located in low- to very low-SES neighborhoods. The lack of statistical significance for alcohol measures may also be due to the fact that we did not compare stores in high SES communities to stores in low SES communities; that feature of our analysis limited variation, and therefore also statistical power, although it offered the advantage of controlling for community SES. However, there may be granular levels of correlation among certain neighborhood characteristics (such as income level, demographic data, and level of neighborhood support or criticism of alcohol sales) and alcohol sales. In our future research, we would like to investigate whether or not that is the case. Future research to examine why stores like DVS choose not to sell alcohol when it is legal to do so would also be useful, particularly as that choice may be associated with local patterns of food consumption or tobacco use.

Neighborhoods with higher proportions of children had higher sales of unhealthy snacks. The majority of children (87%) and adults (87%) report snacking each day; other research has found that American adults eat over 500 cal per day while snacking [44, 47]. It may be that the unhealthy snack foods assessed in this study appealed to children more than adults. For instance, from previous research we are aware that the majority of snack calories that children consume are from desserts, sweets, and salty foods [47]. Research looking at the snacking calories consumed by adults includes alcohol [44], which in the current study was captured in a separate outcome variable.

Also, there was a negative relationship between the percentage of the population that was non-Hispanic African American and tobacco sales [57]. Previous studies found that tobacco outlet density increased as the proportion of African Americans increased [30], and that residents in areas with more tobacco outlets were more likely to smoke [32]. Other researchers found that in some racially integrated neighborhoods White residents were more likely to smoke than African American residents, and that these behaviors can be explained by the social environment [45]. For example, LaVeist et al. [45] found that when whites and African Americans lived in similar conditions, health disparities either decreased, or completely disappeared. The authors concluded that there may be few racial disparities when social factors are equalized [45].

### Limitations

Our results were based on sales at only 16 stores. However, researchers have rarely had access to detailed proprietary data such as the data we used, and our time series data were quite rich with weekly sales over 18 months. This study represented deprived areas, primarily in the Southeastern United States; the findings may not apply to less deprived areas or other regions, or to areas with more racial and socioeconomic diversity. However, the demographic characteristics of the neighborhoods we studied were similar to other low income neighborhoods throughout the Southeast. The data indicated whether products were purchased but did not confirm that the products were consumed. Also, individuals who make purchases at DVS may not live in the same census block group. It is quite possible that individuals who live and work in neighborhoods with a different ADI purchased the unhealthy products studied in this analysis.

## Conclusions

We examined the relationship between neighborhood disadvantage and the sales of tobacco, alcohol, and unhealthy snack food in 16 discount variety stores in the United States. We found that as neighborhood disadvantage increased the units of tobacco products and unhealthy snack foods sold increased. We did not find a difference in alcohol sales by neighborhood disadvantage. Although there could be selection mechanisms and reverse causation between health behaviors and area deprivation, in this study the goods sold in the DVS stores were consistent across neighborhoods except for alcohol. Thus, there was no evidence that the products offered for sale, including tobacco products, varied in response to variation in the characteristics of area populations. People living in disadvantaged neighborhoods are at higher risk for chronic disease and premature death [2, 3]. Greater sales of tobacco products and unhealthy snack foods in disadvantaged neighborhoods may help explain the pathway between neighborhood deprivation and these poor health outcomes. This is the first study to use purchasing data to examine the relationship between neighborhood deprivation and the sales of alcohol, tobacco, and unhealthy snack foods products. It would be useful for future research to better understand the social and environmental factors that influence the sales of tobacco and unhealthy snack foods in deprived neighborhoods.

## Data Availability

The data used are proprietary and are not openly available. Public access to the database is closed.

## Abbreviations

SES: Socio-economic status
ADI: Area Deprivation Index
DVS: Discount Variety Store
ACS: US Census American Community Survey
MELR: Mixed Effects Linear Regression
VIF: Variance Inflation Factors
IQR: Interquartile Range
SNAP: Supplemental Nutrition Assistance Program

## References

[1] Geronimus AT, Bound J, Waidmann TA, Colen CG, Steffick D. Inequality in life expectancy, functional status, and active life expectancy across selected black and white populations in the United States. Demography. 2001;38(2):227–51. Available from: 10.1353/dem.2001.0015. 10.1353/dem.2001.0015 11392910

[2] Clarke CA, Miller T, Chang ET, Yin D, Cockburn M, Gomez SL (2010). Racial and social class gradients in life expectancy in contemporary California. Soc Sci Med.

[3] Seabrook JA, Avison WR (2012). Socioeconomic status and cumulative disadvantage processes across the life course: implications for health outcomes. Can Rev Sociol.

[4] Kind AJ, Buckingham WR (2018). Making neighborhood-disadvantage metrics accessible—the neighborhood atlas. N Engl J Med.

[5] Krieger N, Chen JT, Waterman PD, Soobader MJ, Subramanian SV, Carson R (2002). Geocoding and monitoring of US socioeconomic inequalities in mortality and cancer incidence: does the choice of area-based measure and geographic level matter? The public health disparities geocoding project. Am J Epidemiol.

[6] Krishnan S, Cozier YC, Rosenberg L, Palmer JR (2010). Socioeconomic status and incidence of type 2 diabetes: results from the black Women's health study. Am J Epidemiol.

[7] McGrath JJ, Matthews KA, Brady SS (2006). Individual versus neighborhood socioeconomic status and race as predictors of adolescent ambulatory blood pressure and heart rate. Soc Sci Med.

[8] Solomon CA, Laditka SB, Forthofer M, Racine EF (2019). Black-white disparities in alcohol consumption trends among women in the United States, 1990–2015. J Ethn Subst Abus.

[9] Fischer MJ, Kmec JA (2004). Neighborhood socioeconomic conditions as moderators of family resource transmission: high school completion among at-risk youth. Sociol Perspect.

[10] Franco M, Roux AV, Glass TA, Caballero B, Brancati FL (2008). Neighborhood characteristics and availability of healthy foods in Baltimore. Am J Prev Med.

[11] Kirby JB, Kaneda T (2005). Neighborhood socioeconomic disadvantage and access to health care. J Health Soc Behav.

[12] Lovasi GS, Neckerman KM, Quinn JW, Weiss CC, Rundle A (2009). Effect of individual or neighborhood disadvantage on the association between neighborhood walkability and body mass index. Am J Public Health.

[13] Gordon-Larsen P, Nelson MC, Page P, Popkin BM (2006). Inequality in the built environment underlies key health disparities in physical activity and obesity. Pediatrics..

[14] Singh GK (2003). Area deprivation and widening inequalities in US mortality, 1969–1998. Am J Public Health.

[15] University of Wisconsin School of Medicine Public Health. Area Deprivation Index v2.0. 2019 [cited 2019 May 23]. Available from: https://www.neighborhoodatlas.medicnie.wisc.edu/.

[16] Perrin R. Research by Dr. Amy Kind helps target federal diabetes intervention program. Vital signs: news from the department of medicine. University of Wisconsin-Madison. 2017 Feb 27 [cited 2019 May 23]. Available from: https://vitalsigns.medicine.wisc.edu/research-dr-amy-kind-helps-target-federal-diabetes-intervention-program.

[17] Bhardwaj, R.; Amiri, S.; Buchwald, D.; Amram, O. Environmental Correlates of Reaching a Centenarian Age: Analysis of 144,665 Deaths in Washington State for 2011–2015. Int. J. Environ. Res. Public Health 2020, 17, 2828. Available from: 10.3390/ijerph17082828. 10.3390/ijerph17082828 PMC7215466 32325978

[18] Sheets LR, Kelley LE, Scheitler-Ring K, Petroski GF, Barnett Y, Barnett C, Kind AJ, Parker JC (2020). An index of geospatial disadvantage predicts both obesity and unmeasured body weight. Prev Med Rep.

[19] Kind AJ, Jencks S, Brock J, Yu M, Bartels C, Ehlenbach W, Greenberg C, Smith M (2014). Neighborhood socioeconomic disadvantage and 30 day rehospitalizations: an analysis of Medicare data. Ann Intern Med.

[20] Hu J, Kind AJ, Nerenz D (2018). Area deprivation index predicts readmission risk at an urban teaching hospital. Am J Med Qual.

[21] Singh GK, Miller BA, Hankey BF (2002). Changing area socioeconomic patterns in US cancer mortality, 1950–1998: part II—lung and colorectal cancers. J Natl. Cancer Inst.

[22] Chamberlain AM, Rutten LJ, Wilson PM, Fan C, Boyd CM, Jacobson DJ, et al. Neighborhood socioeconomic disadvantage is associated with multimorbidity in a geographically-defined community. BMC public health. 2020 Dec;20(1):1–0. Available from:https://bmcpublichealth.biomedcentral.com/track/pdf/10.1186%2Fs12889-019-8123-0 10.1186/s12889-019-8123-0 PMC6945427 31906992

[23] U.S. Department of Health and Human Services. The Health Consequences of Smoking – 50 Years of Progress – A Report of the Surgeon General. Atlanta, GA: HHS, CDC, NCCDPHP, OSH; 2014. Available from: https://www.cdc.gov/tobacco/data_statistics/sgr/50th-anniversary/index.htm

[24] U.S. Department of Health and Human Services. Facing Addiction in America: The Surgeon General’s Report on Alcohol, Drugs, and Health. Washington, DC: HHS; 2016. Available from: https://addiction.surgeongeneral.gov/. 28252892

[25] Popkin BM, Duffey K, Gordon-Larsen P (2005). Environmental influences on food choice, physical activity and energy balance. Physiol Behav.

[26] World Health Organization. Noncommunicable diseases. [Internet]. WHO; 2018 [cited September 2020]. Available from: https://www.who.int/en/news-room/fact-sheets/detail/noncommunicable-diseases

[27] Krueger PM, Chang VW (2008). Being poor and coping with stress: health behaviors and the risk of death. Am J Public Health.

[28] Pampel FC, Rogers RG (2004). Socioeconomic status, smoking, and health: a test of competing theories of cumulative advantage. J Health and Soc Behav.

[29] Stimpson JP, Ju H, Raji MA, Eschbach K (2007). Neighborhood deprivation and health risk behaviors in NHANES III. Am J Health Behav.

[30] Lee JGL, Sun DL, Schleicher NM, Ribisl KM, Luke DA, Henriksen L (2017). Inequalities in tobacco outlet density by race, ethnicity and socioeconomic status, 2012, USA: results from the ASPiRE study. J Epidemiol Community Health.

[31] Datta GD, Subramanian SV, Colditz GA, Kawachi I, Palmer JR, Rosenberg L (2006). Individual, neighborhood, and state-level predictors of smoking among US black women: a multilevel analysis. Soc Sci Med.

[32] Pearce J, Rind E, Shortt N, Tisch C, Mitchell R (2015). Tobacco retail environments and social inequalities in individual-level smoking and cessation among Scottish adults. Nicotine Tob Res.

[33] Gruenewald PJ, Remer L, Lipton R (2002). Evaluating the alcohol environment: community geography and alcohol problems. Alcohol Res Health.

[34] American Public Health Association. Addressing alcohol-related harms: a population level response. Policy No. 201912. [internet] APHA; 2019 [updated 2019 5; cited 2020 June]. Available from: https://apha.org/policies-and-advocacy/public-health-policy-statements/policy-database/2020/01/14/addressing-alcohol-related-harms-a-population-level-response.

[35] Ahern J, Galea S, Hubbard A, Midanik L, Syme SL (2008). “Culture of drinking” and individual problems with alcohol use. Am. J. Epidemiol.

[36] Ahern J, Balzer L, Galea S (2015). The roles of outlet density and norms in alcohol use disorder. Drug Alcohol Depend.

[37] Lucan SC, Karpyn A, Sherman S (2010). Storing empty calories and chronic disease risk: snack-food products, nutritive content, and manufacturers in Philadelphia corner stores. J Urban Health.

[38] Ghosh-Dastidar B, Cohen D, Hunter G, Zenk SN, Huang C, Beckman R, Dubowitz T (2014). Distance to store, food prices, and obesity in urban food deserts. Am J of Prev Med.

[39] Richardson AS, Arsenault JE, Cates SC, Muth MK. Perceived stress, unhealthy eating behaviors, and severe obesity in low-income women. Nutr J. 2015;14(1):122. Available from: 10.1186/s12937-015-0110-4. 10.1186/s12937-015-0110-4 PMC4668704 26630944

[40] Walk score: Cities & neighborhoods [Internet]: Walk Score; c2020. [cited 2020 Sept]. Available from https://www.walkscore.com/cities-and-neighborhoods/.

[41] Farley TA, Rice J, Bodor JN, Cohen DA, Bluthenthal RN, Rose D (2009). Measuring the food environment: shelf space of fruits, vegetables, and snack foods in stores. J Urban Health.

[42] Caetano R, Clark CL, Tam T (1998). Alcohol consumption among racial/ethnic minorities: theory and research. Alcohol.

[43] Lariscy JT, Hummer RA, Rath JM, Villanti AC, Hayward MD, Vallone DM (2013). Race/ethnicity, nativity, and tobacco use among US young adults: results from a nationally representative survey. Nicotine Tob Res.

[44] Dunford EK, Popkin BM (2017). Disparities in snacking trends in US adults over a 35 year period from 1977 to 2012. Nutrients..

[45] LaVeist T, Pollack K, Thorpe R, Fesahazion R, Gaskin D (2011). Place, not race: disparities dissipate in southwest Baltimore when blacks and whites live under similar conditions. Health Aff.

[46] Piernas C, Popkin BM (2010). Trends in snacking among US children. Health Aff.

[47] Dunford EK, Popkin BM (2018). 37 year snacking trends for US children 1977–2014. Pediatr Obes.

[48] Riekert KA, Ockene JK, Pbert L (2013). The handbook of health behavior change.

[49] Walk score: Data services [Internet]. Walk Score; c2020. Walkability, real estate, and public health data; [cited 2020 Sept]; Available from: https://www.walkscore.com/professional/research.php

[50] U.S. Census Bureau. Quick facts United States [Internet]. U.S. Census Bureau; 2019 [updated 2019; cited 2020 Sept]. Available from: https://www.census.gov/quickfacts/fact/table/US/PST045219

[51] U.S. Census Bureau. 2019 Population estimates by age, sex, race and Hispanic origin [internet]. U.S. Census Bureau; 2019 [updated 2019, cited 2020 sept]. Available from https://www.census.gov/newsroom/press-kits/2020/population-estimates-detailed.html

[52] Henriksen L, Schleicher NC, Dauphinee AL, Fortmann SP (2011). Targeted advertising, promotion, and price for menthol cigarettes in California high school neighborhoods. Nicotine Tob Res.

[53] Lee JG, Henriksen L, Rose SW, Moreland-Russell S, Ribisl KM (2015). A systematic review of neighborhood disparities in point-of-sale tobacco marketing. Am J Public Health.

[54] Tsourtos G, Ward PR, Muller R (2008). Smoking and stress. Australas Medical J.

[55] Crittenden KS, Manfredi C, Cho YI, Dolecek TA (2007). Smoking cessation processes in low-SES women: the impact of time-varying pregnancy status, health care messages, stress, and health concerns. Addict Behav.

[56] Drewnowski A (2009). Obesity, diets, and social inequalities. Nutr Rev.

[57] Osman A, Kowitt SD, Ranney LM, Heck C, Goldstein AO (2018). Trends and racial disparities in mono, dual, and poly use of tobacco products among youth. Nicotine Tob. Res.

